# Retinoschisis transposition following a retinal detachment repair

**DOI:** 10.3205/oc000029

**Published:** 2015-09-15

**Authors:** Katherine McVeigh, Johannes Keller, Richard J. Haynes

**Affiliations:** 1 Retina Unit, Bristol Eye Hospital, Bristol University Hospitals NHS Trust, Bristol, United Kingdom

**Keywords:** retinoschisis, retinal detachment, vitrectomy, transposition

## Abstract

**Objective:** The authors have observed this phenomenon of translocation of the schisis cavity in a few previous cases and aim to report this unusual finding.

**Method:** A patient with known superotemporal retinoschisis developed a distinctly separate inferotemporal retinal detachment in his left eye. This was repaired with a vitrectomy, cryotherapy and C2F6 tamponade under local anaesthetic. Following surgery, the retinoschisis was found in the inferonasal quadrant of the eye and remained stable as the gas dispersed.

**Result:** We hypothesise that the tamponading agent compressed the viscous fluid within the area of schisis, displacing the area of schisis circumferentially.

**Conclusion:** This case emphasises that as long as the retinal breaks are properly sealed, no intervention is required with the schisis during rhegmatogenous retinal detachment surgery.

## Introduction

Degenerative retinoschisis (RS) is a common condition characterised by the splitting of the retinal layers, occurring between the outer plexiform and inner nuclear layer, creating a cavity filled with hyaluronidase-sensitive acid mucopolysaccharide presumably hyaluronic acid ([1], p. 422) leading to elevation of the inner leaf often in a tense balloon-like configuration. This tends to arise from areas of peripheral microcystoid degeneration and occurs more commonly in hypermetropes, usually affecting the inferotemporal quadrants. Degenerative retinoschisis is found in 5% of the population. Rarely, RS may progress to a rhegmatogenous retinal detachment (RRD) if holes develop in both the outer and inner leafs allowing fluid to pass from the vitreous cavity into the subretinal space [2], [3]. This condition is then treated following the principles of RRD repair. 

## Case description

**Figure 1:**
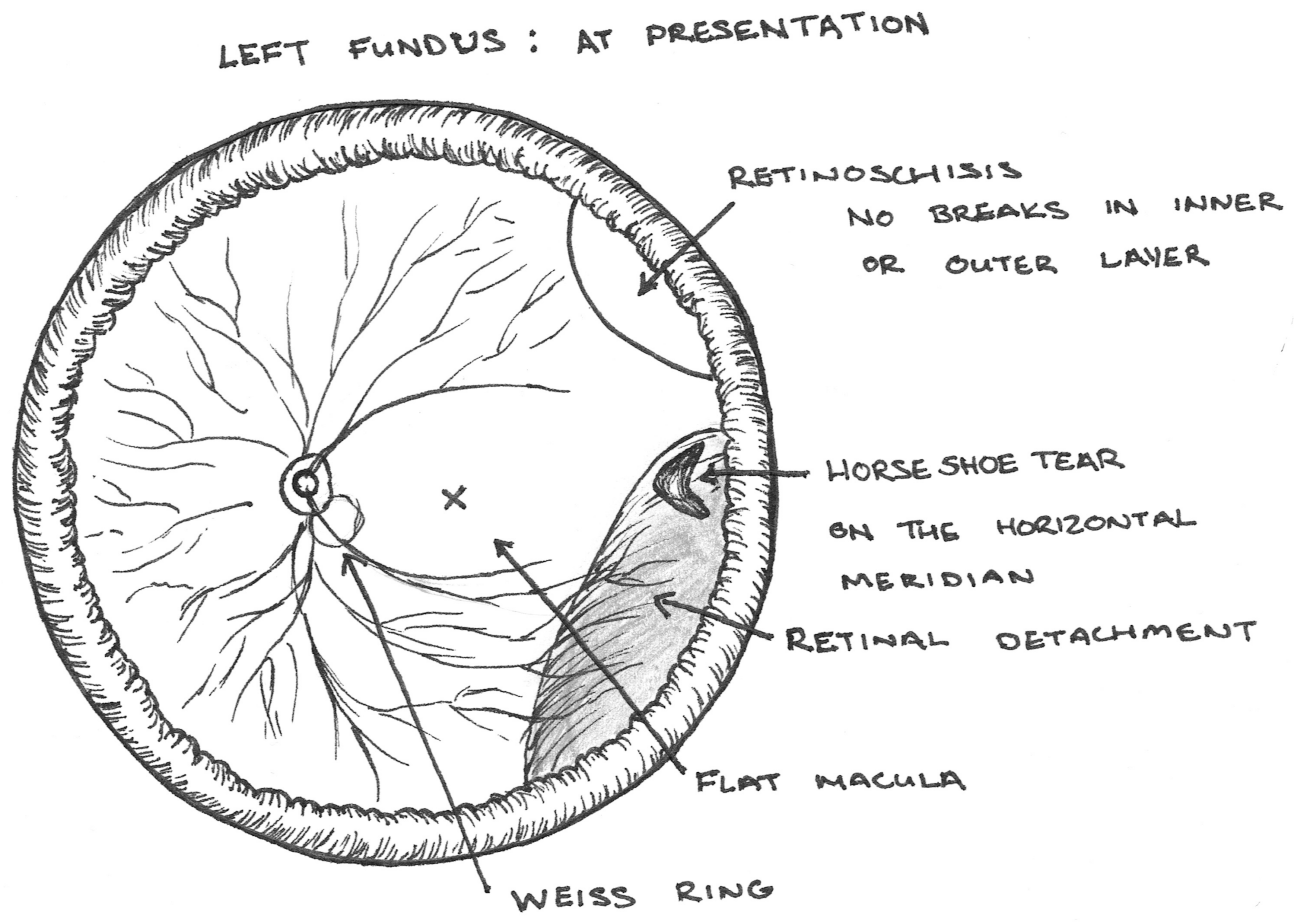
Pre-operative fundal appearance

A 72-year-old patient, previously diagnosed with RS, presented to the emergency clinic complaining of floaters, photopsia and a visual field defect in the left eye. Examination revealed a macula-on inferotemporal RRD with associated posterior vitreous detachment, originating form a horseshoe tear on the horizontal meridian. A distinctly separate area of RS was found superotemporally with no identifiable retinal breaks in either the inner or outer layer on indentation examination (Figure 1 (Fig. 1)). 

The following day the patient underwent pars plana vitrectomy, cryotherapy and internal tamponade with C2F6. Flattening of the detached retina was achieved intraoperatively with perflourodecalin heavy liquid, yet the schitic area remained formed. Prone posturing on the opposite side of the break after logrolling was instructed and supervised by the nursing staff. Postoperatively, an 80% gas fill was achieved and a retinal elevation with an appearance in keeping with RS was noted inferonasally. 

**Figure 2:**
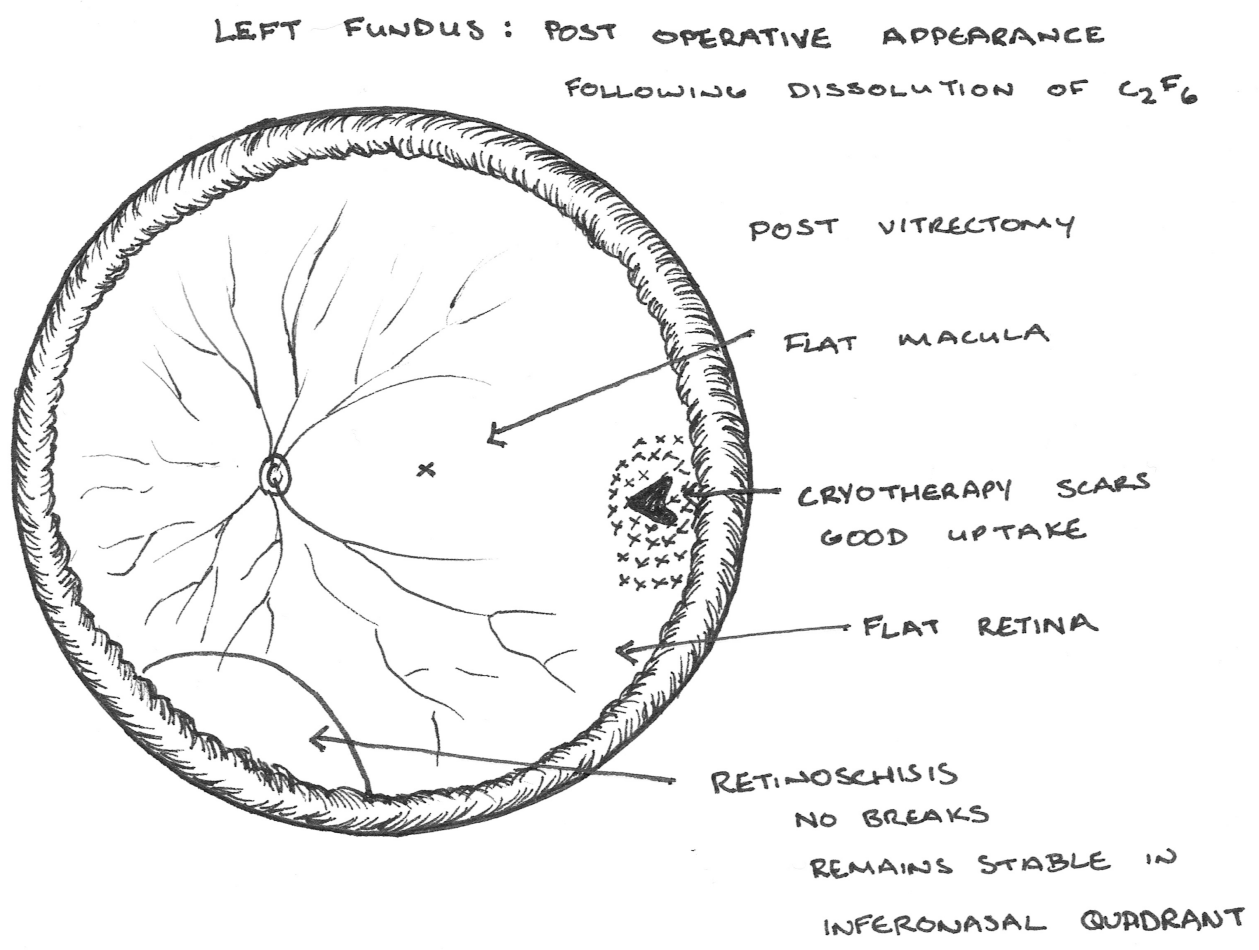
Post-operative fundal appearance

One week later the visual acuity was 6/12 with a 70% gas fill and a flat retina except for the persistence of the raised area of RS inferonasally. Upon full reabsorption of the gas the retina remained flat with an adequate retinopexy scar and the visual acuity improved to 6/9. The RS remained stabilised in the inferonasal quadrant with no further changes (Figure 2 (Fig. 2)).

## Discussion

We present a case in which a pre-existing RS was found to have translocated inferiorly following otherwise uncomplicated RRD surgery. We hypothesise that following fluid-air exchange and subsequent tamponading gas injection, the relative higher density of the schitic fluid within the schisis cavity compared to the gas in the vitreous cavity, resulted in migration of the schitic fluid under the influence of gravity to the most dependent location, in much the same way as sub-retinal fluid will migrate to the most dependent position in a gas filled eye. However, it is hypothesised that in this case the intra-retinal schitic fluid migrated ‘horizontally’ within the outer plexiform layer plane to the most dependent position below the gas bubble. It is postulated that this migration is possible because the underlying abnormality or ‘weakness’ within the outer plexiform layer plane that causes retinoschisis to form in the first place is probably present throughout the peripheral retina and if sufficient force is applied to the viscous fluid within this plane, it will ‘dissect’ its way laterally within the cleavage plane between the outer plexiform and inner nuclear layers until the force is no longer applied i.e. when the schitic fluid has migrated away from the source of the motive force (below the gas bubble).

Accepting that there is potential for the post vitrectomy gas tamponade to induce a change in position of the RS, patients with RS could be asked about previous subjective field defects (although patients are not usually aware of such field defects) and visual field testing could be considered pre surgery to gain an objective measure of the size of defect. In this case, visual field testing pre and post surgical intervention could be used to confirm the transposition of a visual field defect related to the schisis from the inferonasal quadrant to the superotemporal quadrant. However, the patient was not subjectively aware of any schisis-related field defects prior to surgery. The RS did not display breaks in either leaf and it was noted to be an entirely separate entity to the RD. A PVD was present and it was questionable in this case as to whether a buckle would have adequately supported the tear, hence a vitrectomy was performed. 

Although a stable RS is not usually treated with a scleral buckle, this could be considered as a treatment option instead of vitrectomy in cases where the patient or surgeon had concerns regarding the schisis, if breaks were situated anteriorly and in both leaflets of the schisis resulting in a rhegmatogenous schisis detachment. 

There may be doubt whether the post-operative appearance indicates failure or recurrence of the detachment. Close observation or specific testing for schisis may be performed in these cases to decide the course of action. Indentation of the raised area characteristically leads to a tense elevation of a schisis and outer leaf whitening whereas a flaccid flattening would occur with a retinal detachment due to forced evacuation of the sub-retinal fluid from beneath the neurosensory retina via the breaks in a RRD. 

Alternatively a laser test can be used to distinguish a retinal detachment from a retinoschisis. In this test a titrated application of laser to the outer leaf of a schisis leads to whitening of the outer leaf due to the coagulation of the thin layer of outer leaf retina adjacent to the RPE, by contrast no such whitening occurs in the case of a retinal detachment due to the absence of the retinal tissue adjacent to the RPE in a RRD [3]. 

## Conclusion

Transposition of a retinoschisis may occur following a vitrectomy with use of an internal gas tamponade. The proposed mechanism is ‘horizontal’ fluid dissection in the susceptible cleavage plane between the outer plexiform layer and inner nuclear layer, secondary to forces generated by the differential density of schisis fluid compared to gas in the vitreous cavity, leading to migration of the denser schisis fluid to the most dependent location (below the gas bubble) with compression from the gas tamponade and posturing. No additional intervention to the schisis is warranted after surgery as long as new retinal breaks are excluded. 

There were no major concerns noted in this case that would encourage surgeons to strongly consider buckling over vitrectomy when a schisis is present, as this did not affect the surgery or the post-operative outcome. Close monitoring is advised when this is noted, to ensure a further detachment is not overlooked. 

There are several options in dealing with RS associated with a retinal detachment [4]. However, in the case of an incidental finding during retinal detachment repair, and when the spaces are not connected, the RS may be left alone as long as the retinal breaks within the retinal detachment are appropriately treated. 

## Notes

### Competing interests

The authors declare that they have no competing interests.
